# Btk Regulates Macrophage Polarization in Response to Lipopolysaccharide

**DOI:** 10.1371/journal.pone.0085834

**Published:** 2014-01-21

**Authors:** Joan Ní Gabhann, Emily Hams, Siobhán Smith, Claire Wynne, Jennifer C. Byrne, Kiva Brennan, Shaun Spence, Adrien Kissenpfennig, James A. Johnston, Padraic G. Fallon, Caroline A. Jefferies

**Affiliations:** 1 Molecular and Cellular Therapeutics and RCSI Research Institute, Royal College of Surgeons in Ireland, Dublin, Ireland; 2 Trinity Biomedical Sciences Institute, Trinity College Dublin, Dublin, Ireland; 3 School of Biological Sciences, Dublin Institute of Technology, Kevin St, Dublin, Ireland; 4 Institute of Molecular Medicine, St. James’s Hospital, Trinity College Dublin, Dublin, Ireland; 5 National Children’s Research Centre, Our Lady’s Children’s Hospital Crumlin, Dublin, Ireland; 6 Centre for Infection and Immunity, School of Medicine Dentistry and Biomedical Sciences, Queen’s University, Belfast, United Kingdom; 7 Amgen Inc., Thousand Oaks, California, United States of America; Vanderbilt University Medical Center, United States of America

## Abstract

Bacterial Lipopolysaccharide (LPS) is a strong inducer of inflammation and does so by inducing polarization of macrophages to the classic inflammatory M1 population. Given the role of Btk as a critical signal transducer downstream of TLR4, we investigated its role in M1/M2 induction. In Btk deficient (*Btk* ^−\−^) mice we observed markedly reduced recruitment of M1 macrophages following intraperitoneal administration of LPS. *Ex vivo* analysis demonstrated an impaired ability of Btk^−/−^ macrophages to polarize into M1 macrophages, instead showing enhanced induction of immunosuppressive M2-associated markers in response to M1 polarizing stimuli, a finding accompanied by reduced phosphorylation of STAT1 and enhanced STAT6 phosphorylation. In addition to STAT activation, M1 and M2 polarizing signals modulate the expression of inflammatory genes via differential activation of transcription factors and regulatory proteins, including NF-κB and SHIP1. In keeping with a critical role for Btk in macrophage polarization, we observed reduced levels of NF-κB p65 and Akt phosphorylation, as well as reduced induction of the M1 associated marker iNOS in Btk^−/−^ macrophages in response to M1 polarizing stimuli. Additionally enhanced expression of SHIP1, a key negative regulator of macrophage polarisation, was observed in Btk^−/−^ macrophages in response to M2 polarizing stimuli. Employing classic models of allergic M2 inflammation, treatment of *Btk* ^−/−^ mice with either *Schistosoma mansoni* eggs or chitin resulted in increased recruitment of M2 macrophages and induction of M2-associated genes. This demonstrates an enhanced M2 skew in the absence of Btk, thus promoting the development of allergic inflammation.

## Introduction

Macrophages are central players in the development, progression and resolution of inflammation. Similar to the T cell paradigm of Th_1_ and Th_2_ subpopulations, macrophages polarize in response to diverse microbial and environmental signals into various sub-populations with distinct effector functions defined as classic inflammatory M1 and immunosuppressive M2 macrophages. With respect to their role in disease, increased levels of M1 macrophages are associated with autoimmune and inflammatory diseases such as lupus nephritis [1] and multiple sclerosis [2]. M2 macrophages on the other hand have been shown to play a role in promoting tumour growth [3], and in the development of allergic inflammation and airway disease through their ability to induce differentiation of Th_2_ cells (reviewed in [4]).

In recent years much study has gone into understanding the precise molecular mechanism regulating M1/M2 development and polarization. Several studies have implicated key transcription factors and regulatory proteins in this process, including members of the interferon regulatory factor (IRF) family, signal transducer and activator of transcription (STAT) proteins and the suppressors of cytokine signalling (SOCS) family (reviewed in [5]). M1-associated gene induction, following stimulation of macrophages with IFN-γ, LPS or TNFα, is mediated by the activation of STAT1, the p65 subunit of Nuclear factor kappa beta (NF-κB), phosphoinositide 3-kinase (PI3K) and mitogen-activated protein kinases (MAPK), resulting in enhanced production of inflammatory cytokines, chemokines and iNOS [6]–[9]. IL-4 and IL-13 mediate M2 macrophage polarisation by inducing phosphorylation of STAT3 and STAT6 followed by nuclear translocation and M2-associated gene induction [10], [11]. In keeping with the importance of STAT3 and STAT6 in driving M2 macrophage polarization, several studies have demonstrated that inhibition of these proteins promotes an M1 phenotype in macrophages [12]–[14]. Additionally Peroxisome proliferator-activated receptor gamma (PPAR-γ) and Krüppel-like factor 4 (KLF4) have been identified as factors that work in concert with STAT6 to promote an M2 phenotype [15], [16]. Another critical modulator of macrophage polarization is the myeloid restricted Src homology-2 domain-containing inositol 5-phosphatase, (SHIP1), which is an anti-inflammatory protein that functions to convert PIP3 to PI(3,4)P2 in order to turn off PI3K-dependent signalling and negatively regulate NF-κB and IRF3 activity via regulating complex formation and the localisation of key signalling proteins such as TBK1 [17]–[19]. Interestingly, induction of the microRNA miR-155 has been associated with an enhanced M1 phenotype as a result of SHIP1 down regulation [20]. Recently the inhibitory p50 subunit of NF-κB has also been shown to contribute to the process of tolerance and thus M2 macrophage induction by negatively regulating M1 macrophage polarization and IFN-β induction [9]. Furthermore, the suppressors of cytokine signaling (SOCS) proteins also contribute to macrophage polarization, with SOCS3 regulating M1 development while SOCS2 promotes an M2 phenotype [21].

Stimulation of macrophages via Toll Like receptors (TLRs) such as TLR4 or TLR9 has been shown to be a critical signal in driving macrophage polarization via activation of NF-κB or the IRF family members. The Tec tyrosine kinase, Bruton’s tyrosine kinase (Btk), is critical for LPS-induced proinflammatory cytokine production and IFN-γ-induced natural killer cell activation [22]–[26]. Btk interacts with TLR2, 3, 4 and 7 and in doing so mediates their phosphorylation and the transduction of downstream signals. At a molecular level Btk regulates NF-κB activation by regulating p65 phosphorylation downstream of multiple TLRs including TLR4, 7 and 9 [23]–[26]. In addition Btk regulates IRF3 activation downstream of TLR3 and hence IFN-β production in response to viral recognition [27]. Interestingly, IRF3 and IRF5 have been implicated in regulating M1 polarization and associated gene induction [28], [29]. Thus as a critical regulator of transcription factors such as NF-κB and the IRFs, the possibility exists that Btk may regulate macrophage polarization downstream of classical M1and M2 polarizing stimuli.

To fully address this possibility we examined polarisation of macrophages derived from *Btk^−/−^ mice*, which allowed us to directly determine the exact contribution of Btk to this dynamic process. This study shows that Btk plays an important role in regulating LPS-driven M1 polarisation, with impaired recruitment of M1 macrophages and preferential polarisation towards an M2 phenotype observed in the absence of Btk following stimulation with IL-4 and IL-13 or *in vivo* challenge with *Schistosoma mansoni* eggs, a classic model of allergic inflammation. This bias towards an M2 phenotype could not be recovered by treatment with an M1 polarizing cocktail, as this too was shown to promote M2-associated gene induction. At a molecular level, enhanced STAT6 and reduced NF-κB p65, Akt and STAT1 phosphorylation as well as altered SHIP1 induction was shown to contribute to this skew. These studies demonstrate a critical role for Btk in macrophage polarisation with Btk acting as a negative regulator of M2 macrophage induction.

## Materials and Methods

### Ethics Statement

All mouse work was carried out in strict accordance with the requirements of Royal College of Surgeons in Ireland Ethics Committee with ethics approval number: REC582. Animals were culled using CO_2_ asphyxiation and the appropriate organs and cells harvested.

### Reagents

LPS was purchased from Cayla Invivogen (*Escherichia coli* 0111:B4). Recombinant murine IFN-γ, IL-13 and IL-4 were purchased from Immunotools.

### Mice

Btk deficient (*Btk^−/−^*) mice on a C57BL/6 background were a kind gift from Dr Rudi Hendriks [30]. Mice were housed at the Biomedical Research Facility at the Royal College of Surgeons in Ireland under specific pathogen-free conditions. Animals were housed with 12 hour day-night cycle with lights on at 7∶30 pm in a temperature (22±1°C) and humidity (55±5%) controlled room. The animals’ health status was monitored prior to and throughout the experiments and all mice were free of all viral, bacterial, and parasitic pathogens. Prior to commencement of experiments the animals were separated and housed in relevant treatment groups in individually ventilated cages. Each treatment group consisted of 3–4 animals and experiments were performed in triplicate. Prior to and during the experimental period all mice were allowed free access to sterile water and nutrition. All cages contained bedding and were enriched with mouse houses. All efforts were made to minimise suffering.

### Intraperitoneal (i.p) LPS Injection

Age-matched (6 to 10 weeks) wild type (WT) (n = 4) C57BL/6 mice (Harlan Laboratories) and *Btk^−/−^* mice (n = 4) were administered an intraperitoneal injection (i.p.) of 1 mg/kg LPS dissolved in sterile saline solution. Control mice were given an i.p. injection of the equivalent volume of saline. Following 24 hour LPS treatment mice were culled by CO_2_ asphyxiation.

### Isolation of Peritoneal Macrophages

Peritoneal cells were harvested by peritoneal lavage with of 10 ml sterile ice cold PBS. Peritoneal cells were allowed to adhere to plates for 4 hours. Non-adherent cells were subsequently removed by washing with RPMI, and the adherent macrophages were refed with RPMI containing 20% calf serum and gentamicin. Purity of adherent cells (>95%) was determined by flow cytometry following staining with using F4/80 and CD11b (BD Biosciences). Macrophages were used for experiments immediately following isolation. For some experiments macrophages were treated *ex vivo* with an M1 (100 U/ml IFN-γ plus 100 ng/ml LPS) or an M2 (10 ng/ml IL-4 plus 10 ng/ml IL-13) polarizing cocktail for the indicated time points.

### Flow Cytometric Analysis

Peritoneal macrophages were collected from WT or *Btk^−/−^* mice following i.p. LPS injection. Cells were stained for F4/80, CD11b, CD86, and IA/IE (MHCII), using specific antibodies (BD Biosciences) and analysed by flow cytometry using a FACSCantoII flow cytometer (BD Biosciences).

### Real-time PCR

Total RNA was extracted using an RNeasy kit (Qiagen) and reverse transcribed to cDNA using Omniscript reverse transcriptase (Qiagen) according to manufacturer’s recommendations. Quantitative real-time PCR was performed using SYBR Green Taq ReadyMix™ (Sigma) and the data was normalised to a *β-actin* reference. Real-time PCR data was analyzed using the 2^−ΔΔCt^ method [31].

### Helminth Egg Injections

Eggs from *S. mansoni*–infected mice were obtained as described previously [32]. WT mice (n = 4) or *Btk* ^−/−^ mice (n = 4) were given an intravenous (i.v)injection of either 5,000 *S. mansoni* eggs or PBS. Mice were killed on day 14 and lungs were removed. Lungs lobes were snap-frozen or homogenised for analysis of macrophages by flow cytometry or qPCR, as described previously [33].

### In vivo Macrophage Activation

WT mice (n = 4) or *Btk* ^−/−^ mice (n = 4) were injected i.p. with approximately 800 ng Chitin (Sigma) with to induce M2 cells in the peritoneum as previously described [34]. Peritoneal cells were collected by lavage after 48 hours and gene induction was determined by qPCR.

### Western Blot Analysis

WT and Btk^−/−^ BMDMs were treated *ex vivo* with an M1 or M2 polarizing cocktail as indicated. Expression of STAT1(Santa Cruz Biotechnology #sc-592), STAT6 (Santa Cruz Biotechnology #sc-621), pY-STAT1 (Cell Signaling #9171), pY-STAT6 (Imgenex #IMG408A), Akt (Cell Signaling #9271), pS-Akt (Cell Signaling #9272), NF-κB p65 (Santa Cruz Biotechnology #sc-372), pS-NF-κB p65 (Cell Signaling #3036S), iNOS (Transduction Laboratories #N39120) and SHIP-1 (Santa Cruz Biotechnology #sc8425), was determined by Western blot as described previously [23].

### Statistical Analysis

Student’s unpaired *t* test was performed using GraphPad Prism 6.0. Results are presented as mean ± STD. Data were deemed to be significantly different at *P* values less than 0.05.

## Results

### Btk is Required for LPS-elicited Macrophage Polarization in vivo

**Figure 1:**
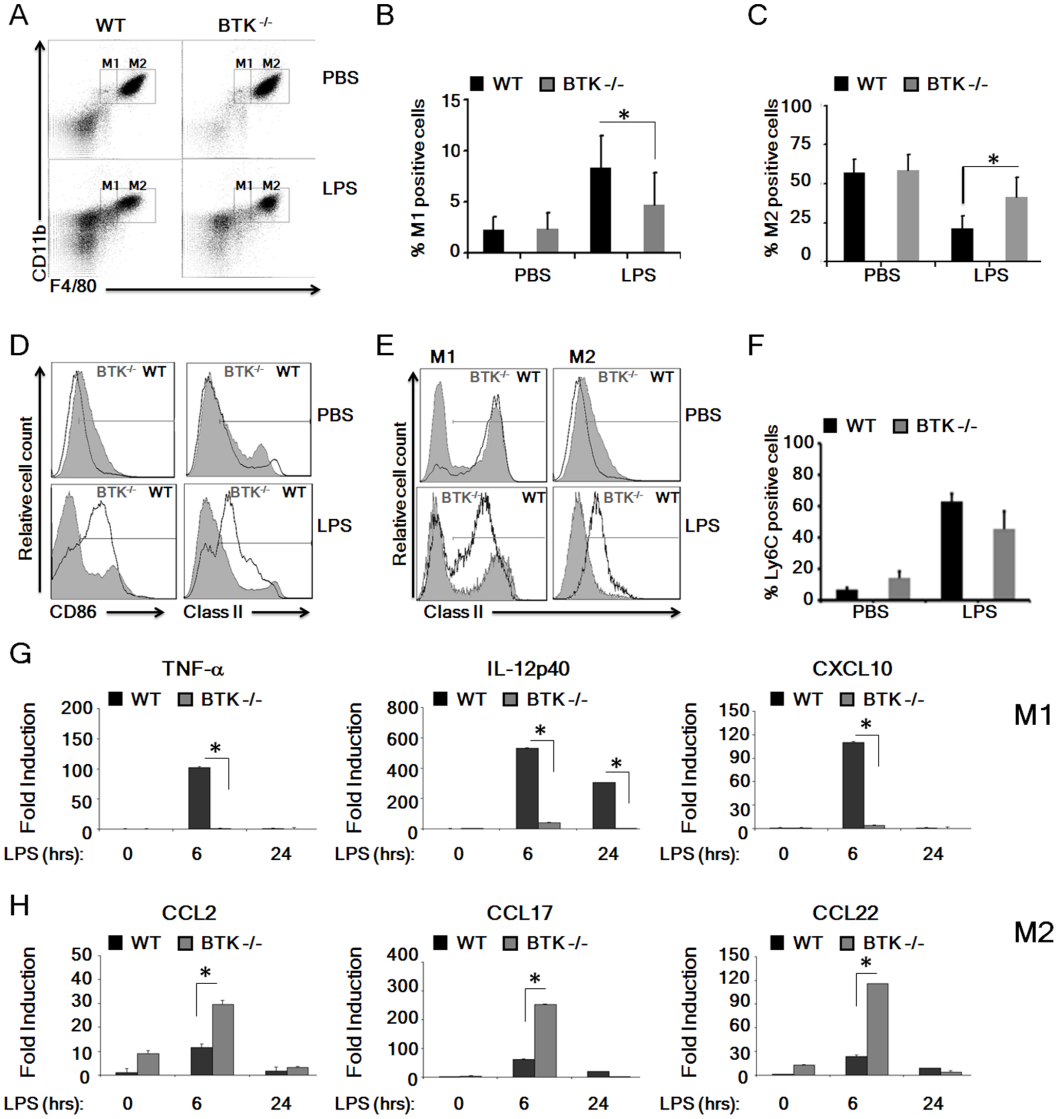
Impaired TLR4-mediated induction of peritoneal M1 cells in *Btk^−/−^* mice *in vivo.* (A–E) Peritoneal macrophages were harvested from WT or *Btk^−/−^* mice following i.p. LPS injection. (A) Representative plots demonstrate the gating strategies for macrophage discrimination where total peritoneal macrophages were defined following co-staining with CD11b APC-Cy7 and F4/80 PE-Cy7. (B–C) Macrophage subsets were further distinguished as F4/80^int^CD11b^hi^ M1-like macrophages and F4/80^hi^CD11b^hi^ M2-like macrophages (M1 and M2 gates, respectively). The relative percentage of M1 (B) and M2 (C) macrophages within the total macrophage gate were determined for WT or *Btk^−/−^* mice following i.p. LPS injection. (D) Expression of CD86 and MHC Class II was determined by flow cytometry. (E) MHC Class II expression was examined within the defined M1 and M2 gates following co-staining with F4/80 and CD11b. (F) Ly6C was determined by flow cytometry. In all cases data is presented as percent increased expression above background as determined by staining with the relevant isotype control (indicated by markers in panel (D). (G–H) Peritoneal macrophages harvested from WT or *Btk^−/−^* mice were treated *ex vivo* with LPS (100 ng/ml) for the indicated time course and the induction of M1- (G) and M2- (H) associated genes was determined by real time PCR (qPCR). Peritoneal macrophages were pooled after isolation, with each treatment group consisting of 3–4 animals, and all experiments were performed in triplicate. Student’s paired *t* test was performed comparing gene induction in Btk^−/−^ peritoneal macrophages to WT cells at the indicated time points. Results shown are mean±SD from three independent experiments. * = p≤0.05.

Given the role of Btk as a central regulator of TLR4-driven pro-inflammatory cytokine production [22], [23], [25], [26], we sought to determine whether Btk was involved in regulating *in vivo* M1/M2 differentiation downstream of TLR4. To date several surface antigens have been employed in order to discriminate between polarized macrophage populations. Initial studies have suggested that size and relative expression of F4/80 and CD11b can distinguish subtypes which are loosely defined as F4/80^int^CD11b^hi^ M1-like macrophages and F4/80^hi^CD11b^hi^ M2-like macrophages [35]. However, while this approach has utility, the authors noted that this technique did not fully address the heterogeneity of the population. It is now therefore accepted that in addition to F4/80 and CD11b the relative expression of antigen presentation molecules, co-stimulatory molecules and Ly6C can be used to more fully discern polarized macrophages, with M1 macrophages demonstrating increased expression of these antigens relative to M2 macrophages [36]–[38]. Taking these approaches into account, LPS was injected i.p. into WT and *Btk* ^−/−^ mice and 24 hours later peritoneal macrophages were profiled by flow cytometry for F4/80 and CD11b (Figure 1A), following which analysis of the relative proportion of F4/80^int^CD11b^hi^ M1-like macrophages and F4/80^hi^CD11b^hi^ M2-like macrophages was performed (Figure 1B and 1C, respectively). Gating on F4/80^int^CD11b^hi^ we assessed the effect of LPS on recruitment of M1-like macrophages to the peritoneum and found that in the absence of Btk significantly reduced levels of F4/80^int^CD11b^hi^ cells (8.5% v 4.8%; p≤0.05) were observed compared to WT mice (Figure 1 B). Additionally we assessed recruitment of M2-like macrophages by gating on F4/80^hi^CD11b^hi^ cells and determined that significantly enhanced levels of these cells (41.5% v 20.9%; p≤0.05) were recruited to the peritoneum in the Btk-deficient mice compared to similarly treated WT mice (Figure 1 C). Consistent with suggestions that that these F4/80^hi^CD11b^hi^ cells represent an influx of M2-like macrophages Btk-deficient peritoneal macrophages displayed reduced levels of the co-stimulatory molecule CD86 (33% v 62%) and reduced expression of MHC class II (39% v 73%) when compared to WT mice (Figure 1 D). In addition, analysis of MHC Class II expression within M1 and M2 gates as defined in Figure 1A revealed that the majority of expression for this antigen was within the F4/80^int^CD11b^hi^ (M1) gated region, with WT peritoneal macrophages displaying enhanced levels of MHC class II when compared to Btk-deficient peritoneal macrophages (Figure 1 E). Furthermore reduced expression of Ly6C was observed on infiltrating myeloid cells following LPS treatment in *Btk^−/−^* mice compared to WT mice (Figure 1 F). This data suggests that in the absence of Btk M2-like macrophages are preferentially recruited following exposure to LPS.

Polarized macrophage subsets can be further distinguished according to the array of cytokines and chemokines they differentially secrete [39], [40]. We investigated gene induction profiles of M1- and M2- associated genes in WT and Btk^−/−^ peritoneal macrophages following *ex vivo* LPS treatment (Figure 1 G–H). As expected, LPS-treated WT peritoneal macrophages displayed significantly enhanced expression of the characteristic M1-associated genes *Tnfα*, *Il12p40* and *Cxcl10* when compared to Btk^−/−^ cells (Figure 1G). In contrast, LPS-treated Btk^−/−^ macrophages had significantly reduced expression of M1-associated genes but enhanced expression of the M2-associated chemokines *Ccl2*, *Ccl17* and *Ccl22* in comparison to WT macrophages (Figure 1H). Thus not only are Btk^−/−^ macrophages defective in their ability to induce proinflammatory cytokines in response to LPS [23]–[26], they appear to preferentially polarize towards anti-inflammatory M2 macrophages in response to this normally pro-inflammatory stimulus.

### Dominant M2 Phenotype in Macrophages Lacking Btk

**Figure 2:**
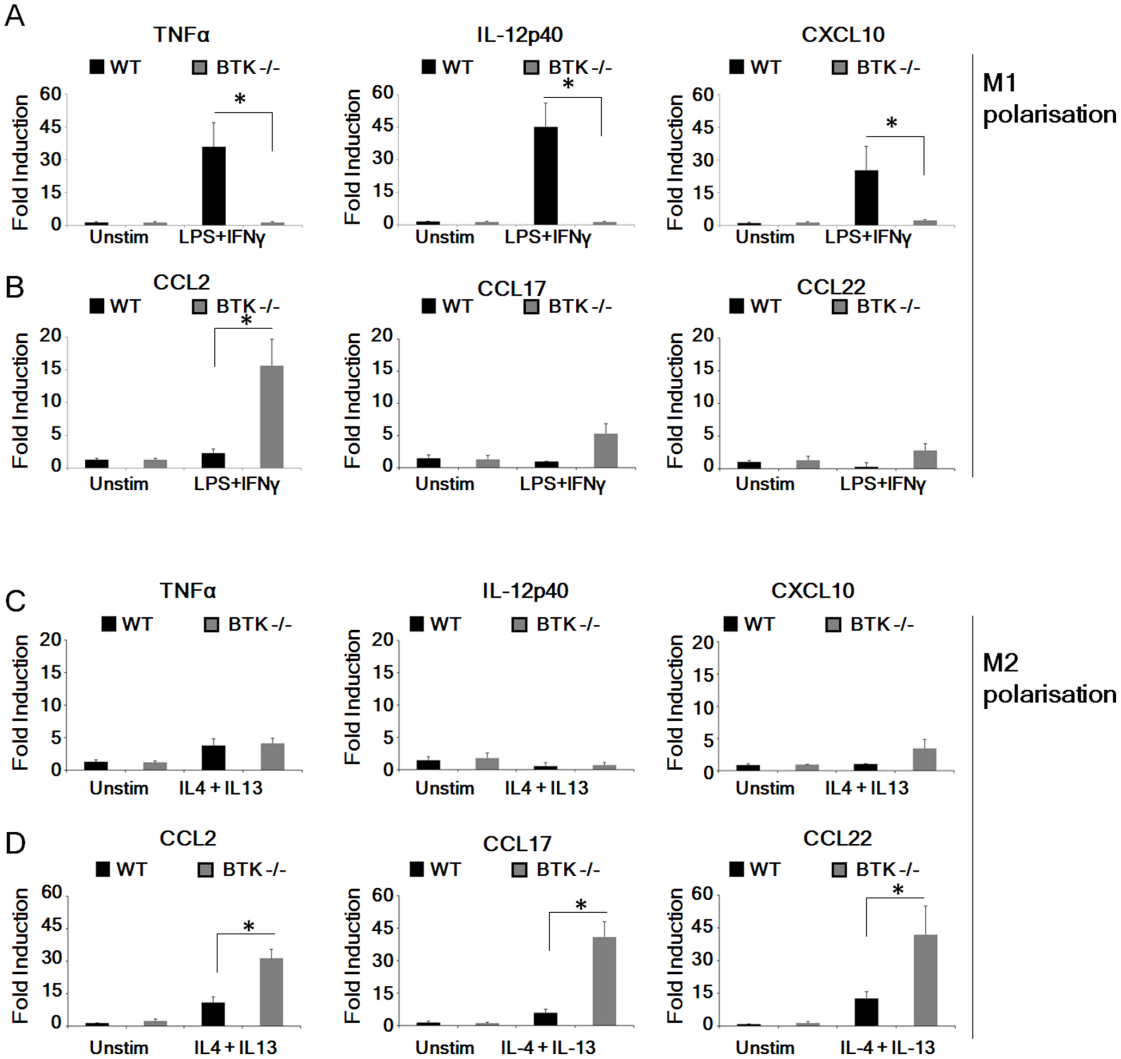
Btk^−/−^ macrophages have impaired ability to expand *in vitro* into M1 cells. (A–D) Peritoneal macrophages were extracted from WT and *Btk^−/−^* mice and treated *ex vivo* with an M1 (LPS plus IFN-y) or an M2 (IL-4 plus IL-13) polarizing cocktail for 24 hr and induction of M1 (A–B) and M2- associated (C–D) genes determined by qPCR. In all cases peritoneal macrophages were pooled after isolation, with each treatment group consisting of 3–4 animals, and all experiments were performed in triplicate. Student’s paired *t* test was performed comparing gene induction in Btk^−/−^ peritoneal macrophages to WT cells following treatment with polarizing cocktails as indicated. Results shown are mean±SD from three independent experiments. * = p≤0.05.

Given that polarized macrophage states can be modulated or reversed [41], we next asked whether Btk^−/−^ macrophages would polarize normally in response to either M1 or M2 polarizing stimuli. Peritoneal macrophages isolated from WT and Btk^−/−^ mice were treated *ex vivo* with either an M1 (LPS plus IFN-γ) or an M2 (IL-4 plus IL-13) polarizing cocktail and gene induction was determined by real time PCR. As expected, treatment with the M1 polarizing cocktail resulted in robust induction of *Tnfα*, *Il12* and *Cxcl10* in WT macrophages (Figure 2 A). However, similarly treated Btk^−/−^ macrophages demonstrated impaired induction of M1-associated genes, instead showing a marked propensity to express M2-associated genes (Figure 2 B). Treatment with the M2 polarizing cocktail resulted in expression of M2-associated genes in WT and Btk^−/−^ macrophages; however Btk^−/−^ macrophages exhibited significantly enhanced induction of M2-associated genes when compared to WT macrophages (Figure 2 D; p≤0.05). Thus our results strongly indicate that Btk^−/−^ macrophages preferentially polarize to an M2 phenotype and that these cells are incapable of switching to an M1 phenotype following exposure to IFN-γ and LPS. Indeed the M2 skew is exacerbated by exposure to M2 polarizing stimuli in the Btk^−/−^ macrophages compared to WT, indicating that pathways governing M2 polarization are constitutively primed in Btk^−/−^ macrophages.

### Altered Phosphorylation of Key Signalling Intermediaries in the Absence of Btk

**Figure 3:**
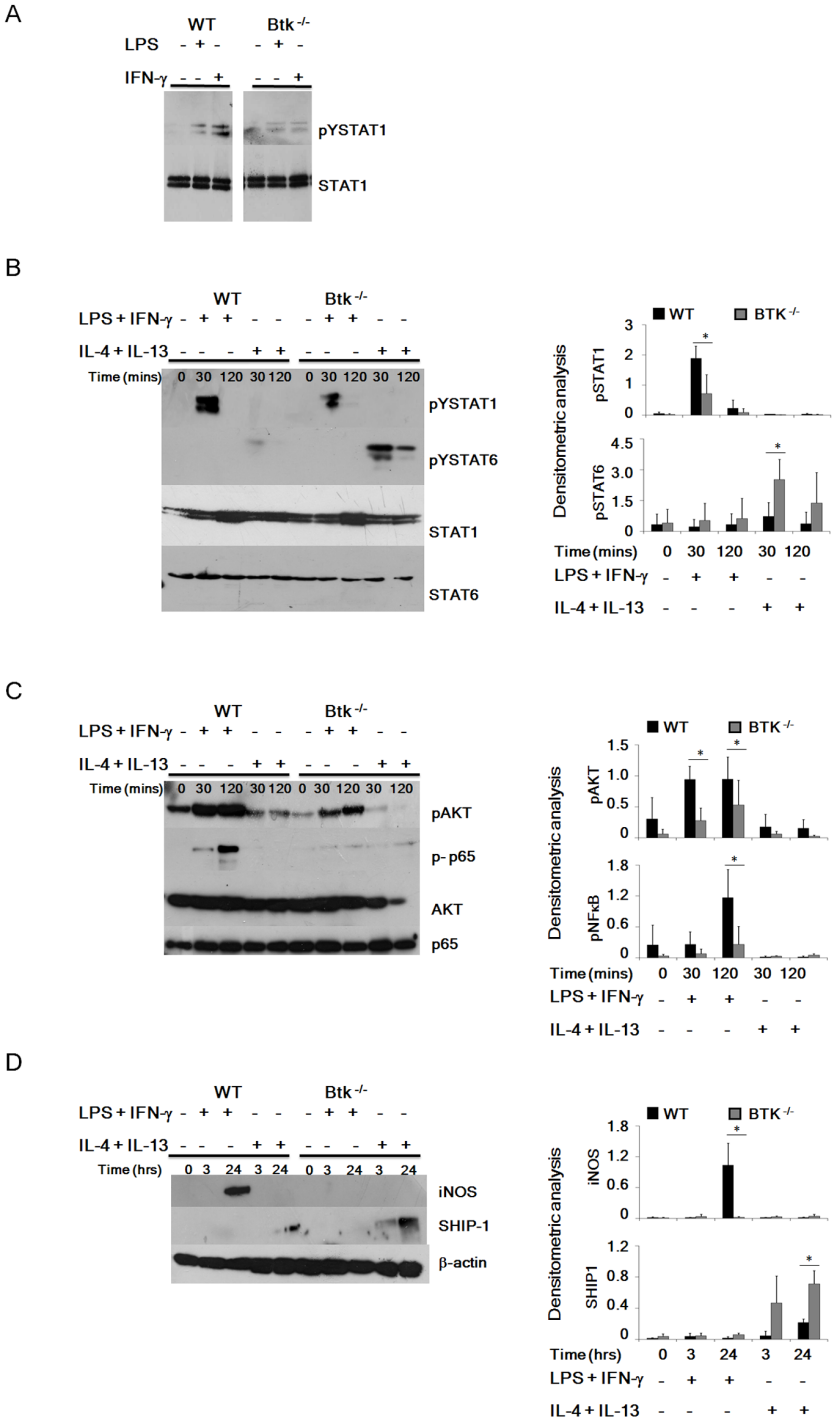
Altered phosphorylation of key signalling intermediaries in the absence of Btk. (A) WT and Btk^−/−^ BMDMs were treated *ex vivo* with LPS (100 ng/ml) or IFN-γ (100 U/ml) for 3 hours or 15 minutes, respectively, lysates were prepared and phosphorylated Y701-STAT1 levels determined by Western blot. WT and Btk^−/−^ BMDMs were treated *ex vivo* with an M1 or M2 polarizing cocktail over the indicated time course, lysates were prepared and tyrosine phosphorylated STAT1 and STAT6 (B), serine phosphorylated AKT (upper panel) and NF-κB p65 (lower panel) (C) iNOS and SHIP-1 (D) levels were determined by Western blot. Results in each case are representative of three independent experiments. Densitometric analysis was performed and graphs represent phosphorylated protein levels relative to unphosphorylated proteins (B-C) or changes in total protein levels relative to β-actin (D) for WT and Btk^−/−^ BMDMs. Student’s paired *t* test was performed comparing relative expression of phosphorylated or total proteins in Btk^−/−^ BMDMs to WT BMDMs following treatment with polarizing cocktails as indicated. Results shown are mean±SD from three independent experiments. * = p≤0.05.

We next sought to investigate the mechanism by which Btk contributes to macrophage polarisation. Investigating the effects of LPS or IFN-γ on STAT1 activation we observed that tyrosine phosphorylation of STAT1 was impaired in the absence of Btk following culture of BMDMs with either LPS or IFN-γ alone (Figure 3 A). Additionally the combination of LPS and IFN-γ also induced less STAT1 phosphorylation in Btk^−/−^ BMDMs compared to WT BMDMs (Figure 3 B). Consistent with increased generation of M2 macrophages in the absence of Btk, we observed enhanced phosphorylation of STAT6 in response to IL-4 and IL-13 in the Btk^−/−^ BMDMs compared to WT BMDMS (Figure 3 B, lower panel).

It is well established that in addition to STAT activation M1 and M2 polarizing signals modulate the expression of inflammatory genes via differential activation of transcription factors, including NF-κB, as a result of activation of key pathways including PI3K/Akt [42]. Not surprisingly given the previously identified role for Btk in regulating p65 phosphorylation and hence NF-κB activation, and the role NF-κB plays in regulating M1/M2 differentiation, we observed significantly less phosphorylation of p65 in Btk^−/−^ BMDMs compared to WT BMDMs following LPS and IFN-γ treatment, whereas M2 polarizing conditions failed to promote p65 phosphorylation either in WT or Btk^−/−^ BMDMs (Figure 3 C). Similarly phospho-Akt levels were also reduced under the same conditions in Btk^−/−^ BMDMs, and again M2-polarizing signals failed to induce Akt activation in either WT or Btk^−/−^ cells. In addition under the same conditions, we observed potent induction of the classical pro-inflammatory *M1-associated* marker *iNOS* in WT BMDMs when compared to Btk^−/−^ BMDMs (Figure 3 D), with M2 polarizing signals having no effect. These results indicate that Btk mediates M1 gene induction via promoting Akt and p65 activation. Regarding what might drive Btk^−/−^ macrophages to become skewed towards an M2 phenotype even in the presence of M1 polarizing signals, SHIP1, as a key anti-inflammatory protein that negatively regulates PI3K-dependent signalling and subsequently activation of NF-κB and IRF3 thus negatively regulating M1 macrophage polarisation, is a potential target [17]–[19]. In keeping with the importance of SHIP1 in promoting M2 polarization, treatment with IL-4 and IL-13 resulted in expression of SHIP1 in both WT and Btk^−/−^ BMDMs, however expression levels were enhanced in Btk^−/−^ macrophages compared to WT macrophages, suggesting inappropriate regulation of SHIP-1 expression and potentially activity in Btk^−/−^ BMDMs contributes to the M2 skew observed (Figure 3 D, lower panel). Thus in the absence of Btk diminished activation of key signaling intermediaries and transcription factors and enhanced expression of the M2-promoting proteins including STAT6 and SHIP1 accounts for the inability of macrophages to effectively polarize towards an M1 phenotype.

### Absence of Btk Exacerbates M2 Recruitment and Induction Following Induction of Allergic Inflammation

**Figure 4:**
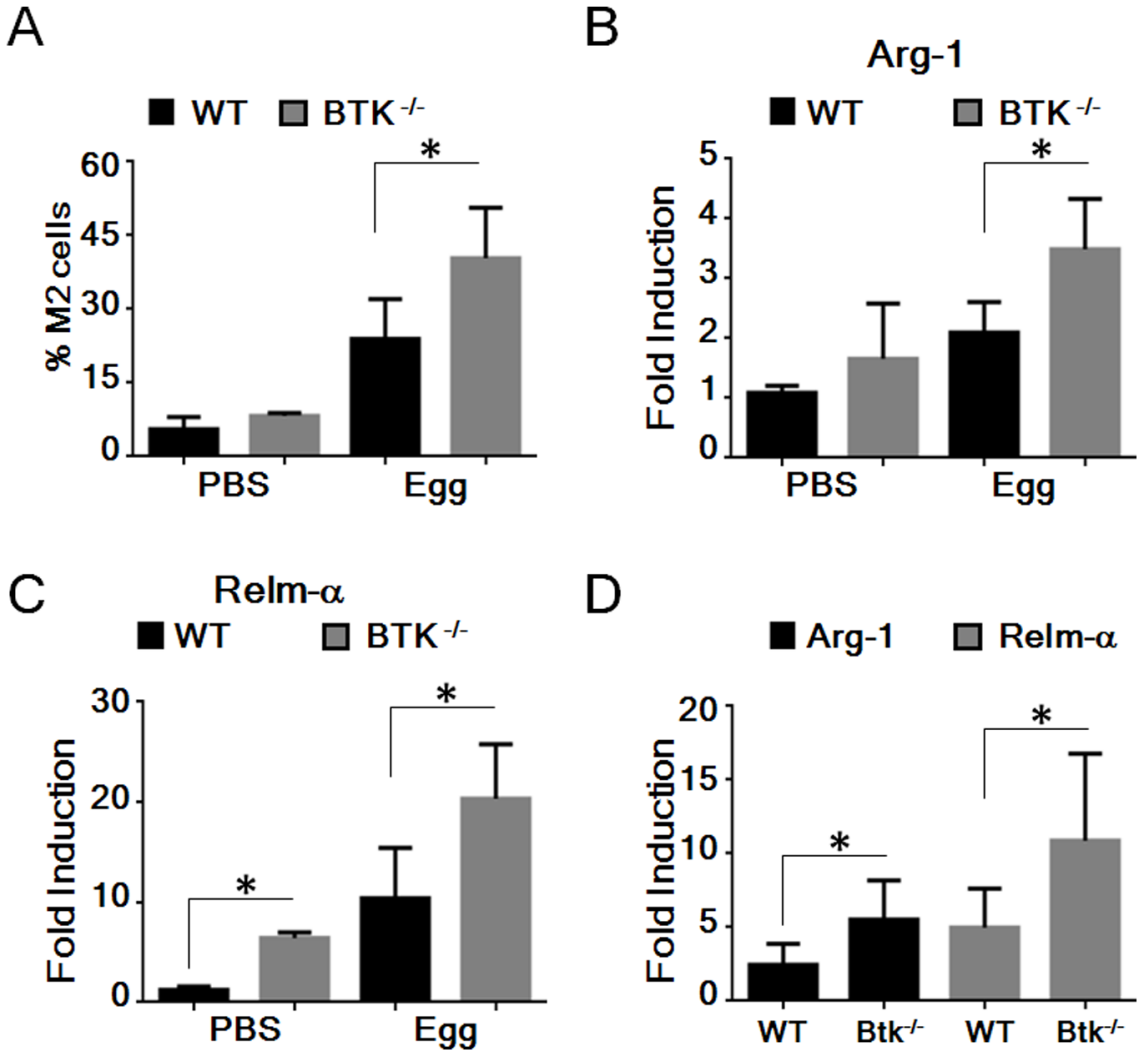
Increased *in vivo* M2 macrophage generation in the absence of Btk. (A–C) WT and *Btk^−/−^* mice were injected i.v. with 5,000 live *S.mansoni* eggs. (A) The percentage of pulmonary M2 macrophages was evaluated by flow cytometry using F4/80 and CD11b co-staining. (B–C) Induction of M2-associated genes was determined by qPCR. (D) WT and *Btk^−/−^* mice were injected i.p. with approximately 800 ng Chitin. Peritoneal cells were collected by lavage after 48 hours and gene induction of M2-associated genes was determined by qPCR. In all cases peritoneal macrophages were pooled after isolation, treatment groups consisted of 3–4 animals, and experiments were performed in triplicate. Student’s paired *t* test was performed comparing gene induction following *in vivo* exposure to *S.mansoni* eggs or Chitin as indicated in WT and Btk^−/−^ peritoneal macrophages. Results shown are mean±SD from three independent experiments. * = p≤0.05.

To date our data suggests that loss of Btk results in a predominant M2 phenotype which may potentially aggravate M2-mediated disease such as allergic inflammation. Both S*chistosoma mansoni* eggs and chitin drive allergic inflammation via promoting M2 induction and a Th_2_-mediated response [43], [44]. We hypothesised that M2 induction would be exacerbated in the absence of Btk. WT and *Btk^−/−^* mice were injected i.v. with 5,000 *S. mansoni* eggs and 14 d later M2 macrophages were analysed in lungs by flow cytometry, with induction of M1 or M2 associated genes determined by qPCR. In keeping with our previous findings, we observed a significant (p≤0.05) increase in recruitment of M2 macrophages to the lungs of *Btk^−/−^* mice compared to WT mice (Figure 4 A), accompanied by a preferential induction of M2-associated genes, Arginase 1 and Relm-α, in *Btk^−/−^* mice compared to WT mice (Figure 4 B and C).

To further address the role of Btk in *in vivo* generation of M1 versus M2 cells, WT and *Btk^−/−^* mice were injected i.p. with chitin following which peritoneal cells were collected by lavage and M1/M2 gene induction determined by qPCR. Similar to *S. mansoni* challenge, chitin treatment of *Btk^−/−^* mice resulted in enhanced induction of the M2-associated genes Arginase 1 and Relm-α, in peritoneal exudate cells compared to controls (Figure 4 D). Similar to the enhanced basal levels of M2-associated genes in the peritoneum following *in vivo* LPS treatment (Figure 1 G) we also observed enhanced basal levels of M2-associated genes *in vivo* (Figure 4 B–D). Collectively, using two separate M2 inducing *in vivo* mouse models, in lungs or peritoneum, there is marked polarization to M2 cells in the absence of Btk.

## Discussion

**Figure 5:**
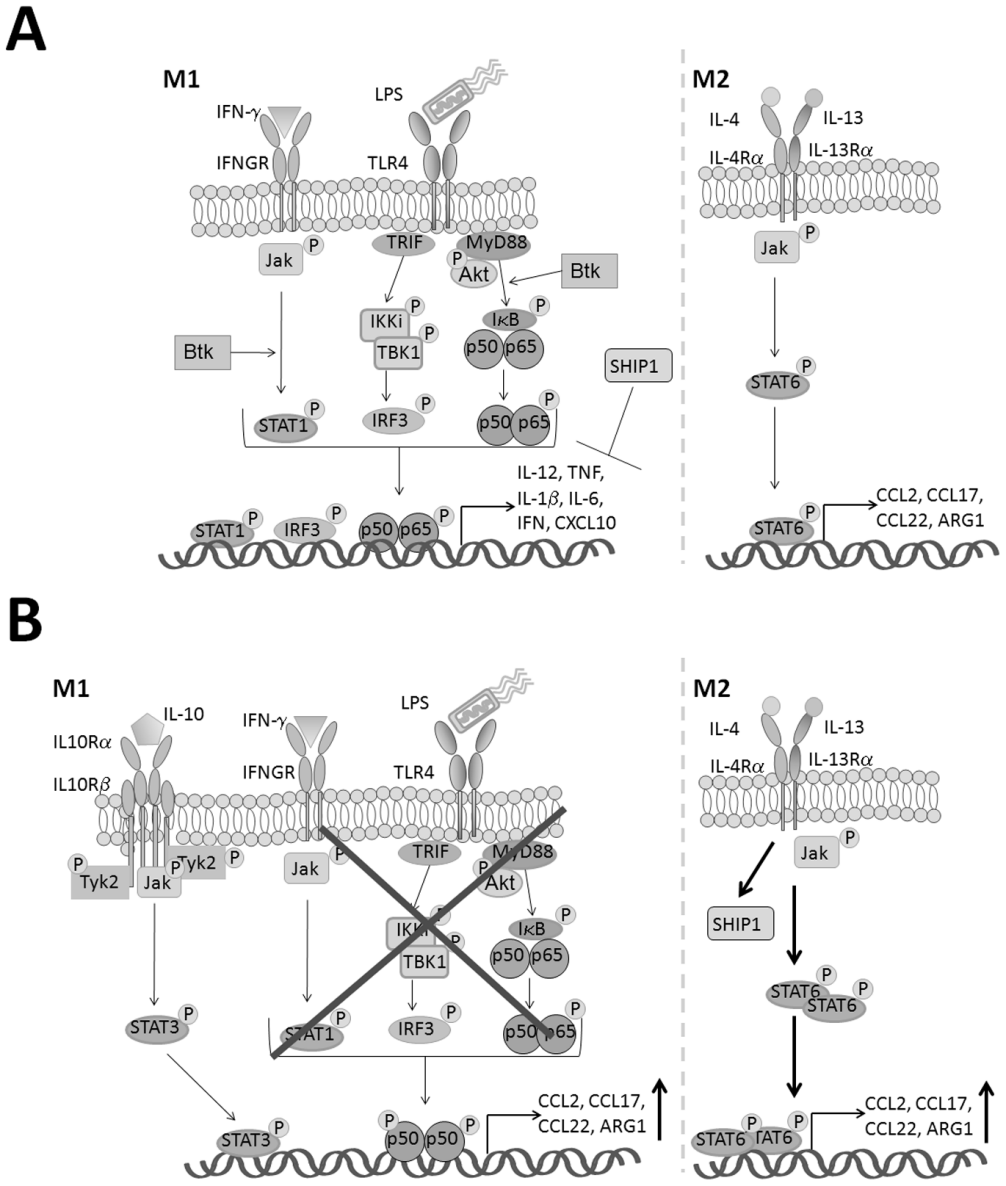
Proposed transcriptional regulation of macrophage polarization in the absence of Btk. (A) This study has demonstrated that in response to LPS and IFN-γ Btk contributes to M1 polarizing of myeloid cells via promoting the phosphorylation of Akt and subsequently the p65 subunit of NFκB, in addition to enhancing to phosphorylation of STAT1. (B) In the absence of Btk exposure of myeloid cells to LPS and IFN-γ results in the preferential induction of M2 associated genes and preferentially recruitment of M2 cells *in vivo*. Previous studies in *Btk^−/−^* mice have observed increased levels IL-10 systemically following LPS treatment. IL-10 is known to activate STAT3 and there is some evidence to suggest that STAT3 may also play a role in promoting M2 macrophage polarization. Thus in the absence of Btk, in response to M1 polarizing stimuli increased IL-10 production together with reduced phosphorylation of key signaling intermediaries, combined with activation of p50 the inhibitory subunit of NF-κB could potentially account for the observed preferential skew towards an M2 phenotype. Additionally this study has shown that in response to IL-4 and IL-13 Btk^−/−^ cells demonstrate an increased capacity to polarize towards an M2 phenotype as a result of enhanced STAT6 phosphorylation and increased SHIP1 expression.

This study demonstrates a novel role for Btk in determining macrophage lineage commitment, with preferential M2 macrophage induction observed in the absence of Btk as a result of altered activation of key signaling pathways and transcription factors such as NF-κB p65, Akt and proteins known to regulate macrophage polarization including SHIP1 and members of the STAT family. Furthermore we show that Btk is essentially required downstream of TLR4 and IFN-γ for optimal p65-dependent and STAT1-dependent M1 macrophage polarization (Figure 5).

To date there has been no direct evidence supporting a role for Btk in macrophage polarization and reports of Btks contribution to inflammation have been controversial. Several studies have suggested that Btk functions as a negative regulator of inflammation [45]–[47] while there is gathering evidence to suggest that Btk may be important in driving the inflammatory process. Studies examining APCs derived from XLA patients, who have a natural mutation in Btk, have shown that they have defects in both phagocytosis and inflammatory cytokine production following TLR stimulation [25], [26], [48]. Recently the potential that Btk plays a direct role in positively regulating antigen presentation and maturation of APCs was described by Lui *et al.* These studies found impaired TLR-driven activation of APCs due to an inability to form a MHC class II and CD40 complex in the absence of Btk [49]. At a molecular levels Btk has been shown to stabilise TNF-α mRNA via a p38- dependent pathway in X-linked immunodeficiency (Xid) mice and in XLA patients as well as being critically involved in NF-κB activation and specifically p65 phosphorylation downstream of multiple TLRs including TLR4, TLR7 and TLR9 [23]–[26]. More recently studies by our group have suggested that increased IL-10 production together with a significant reduction in IFN-γ production in the absence of Btk may result in an altered Th_1_/Th_2_ balance [22], potentially implicating Btk in macrophage polarization. In the current study we observed impaired activation of NF-κB p65 in the absence of Btk following TLR4 and IFN-γ treatment, further supporting a positive role for Btk in M1 macrophage polarisation. Interestingly, M2 macrophages have been shown to inhibit the expression several M1 associated chemokines including CXCL10 by modulating the activity of both NF-κB and STAT1 [50], [51]. We observed impaired phosphorylation of Akt, a key MyD88-dependent signalling intermediary and inducer of M1-associated cytokines [52], [53] in the absence of Btk. Studies in B cells have shown that Btk and Akt directly interact and that in the absence of Btk, Akt activation is prevented [53]. A recent study has implicated Akt as a negative regulator of Btk, phosphorylating it in order to promote 14-3-3ζ binding, a novel negative regulator of Btk signalling [54]. Thus the ability of Btk and Akt to cross-regulate each other and the involvement of Akt in regulating macrophage polarization, indicates that the inability of Btk^−/−^ macrophages to phosphorylate Akt in response to M1 polarizing stimuli has an important contribution to the M2 skew observed in these cells.

Whilst a direct link between Btk and STAT1 in regulating macrophage activity has not been previously demonstrated, overexpression studies and studies in B cells have demonstrated that Btk and other Tec family kinases can interact with STAT family members and promote or inhibit their activation. Btk has been shown to interact directly with JAK1 and this association results in Btk phosphorylation [55]. More recently the absence of Btk was shown to result in reduced polyI:C-mediated activation of STAT1 [27]. Relevant to our findings, Btk has been shown to prevent STAT3 activation in B cells thus promoting apoptosis [56]. Overall our study indicates that Btk is required for optimal STAT1 activation and that in the absence of Btk, enhanced STAT6 phosphorylation is observed, in keeping with the inhibitory role for Btk regarding STAT3 activation reported by Uckun *et al* [56]. Given that Btk has been implicated as key positive regulator of TLR4-mediated cytokine production and that STAT1 activation is required for optimal LPS induced activation of macrophages [57], this study demonstrates an important link between Btk activation, subsequent transcription factor phosphorylation and macrophage polarization.

Interestingly whilst exposure of Btk-deficient macrophages to M1 polarizing stimuli did not result in increased STAT6 phosphorylation or SHIP1 induction, we did however observe significantly impaired activation of key M1 promoting signalling molecules, such as NF-κB p65, STAT1 and AKT, as previously published [6]–[9]. Together our data suggests that not only is Btk critical for the induction of signals driving M1 polarization but that it also functions as a negative regulator of M2-polarizing signalling pathways, as evidenced by the hyper-phosphorylation of STAT6 and increased induction of SHIP1 we observed following IL-4 and IL-13 treatment in the absence of Btk. The possibility therefore exists that much like SHIP1 [58]–[60] and Akt [61], [62], Btk may have a dual function in mediating macrophage polarization. This role is supported by studies demonstrating that Btk phosphorylates the key TLR4 adaptor Mal, resulting in SOCS1-mediated ubiquitination and degradation of Mal and hence negative regulation of TLR4-dependent pathways [7]. Additionally unpublished observations in our group demonstrate that SHIP1 and Btk interact and that this interaction regulates the phosphorylation and presumably the activity of SHIP1 in an as yet undetermined manner. Thus the lack of Btk contributes not only to the expression of SHIP1 but may also play a role in regulating its activity.

Thus our data suggests that loss of Btk results in a predominant M2 phenotype which may potentially exacerbate Th2 mediated disease such as allergic asthma. Asthma is traditionally thought of as a disease mediated by an imbalance between Th_1_/Th_2_/Th_17_ cells, however it is becoming more apparent that alveolar macrophages play an important role in directing disease outcome [2], [4]. Indeed the enhanced production of M2-associated chemokines by alveolar macrophages is thought to contribute to small airway and peripheral lung inflammation observed in asthma patients [63]. Our data in two models of allergic disease demonstrate that a lack of Btk exacerbates M2 polarization, indicating the critical role of this protein in ensuring a balanced response and strongly suggests that lack of a proper M1/M2 balance in the lung in the absence of Btk may exacerbate allergic inflammation. Indeed a study of allergic airway inflammation in mice reports increased IgE responses an exaggerated airway inflammation in the absence of Btk [64]. Clinically, most likely due to absent circulating immunoglobulin and a role for Btk in mast cell degranulation, reports of allergy in XLA patients are rare [65], [66]. Despite the rarity of allergic reactions, pulmonary complications such as decreased lung function and increased thickening have been observed in XLA patients [67]. Given the known role of macrophages in driving airway disease, it is tempting to propose that an M1/M2 imbalance may be driving this response.

Btk inhibitors are currently in trial for a number of conditions including B cell malignancies and inflammatory autoimmune conditions [68], [69]. Whilst undoubtedly Btk inhibitors contribute to reduced production of pro-inflammatory cytokines by macrophages, Btk inhibition also affects apoptotic cell uptake, in addition to its role in regulating macrophage polarization as demonstrated here [70]. Given the emerging role for macrophages in allergy, asthma, cancer and certain autoimmune conditions such as systemic lupus erythematosus, our findings suggest that manipulation of Btk activity may have unwanted effects in certain disease settings and indicates the need for more extensive analysis of the role of Btk in macrophages in inflammatory disease.

## References

[1] Orme J, Mohan C (2012) Macrophage subpopulations in systemic lupus erythematosus. Discov Med 13: 151–158. 22369974

[2] Shechter R, Schwartz M (2013) Harnessing monocyte-derived macrophages to control central nervous system pathologies: no longer ‘if’ but ‘how’. J Pathol 229: 332–346. 23007711 10.1002/path.4106

[3] Hao NB, Lu MH, Fan YH, Cao YL, Zhang ZR, et al (2012) Macrophages in tumor microenvironments and the progression of tumors. Clin Dev Immunol 2012: 948098. 22778768 10.1155/2012/948098 PMC3385963

[4] Dasgupta P, Keegan AD (2012) Contribution of alternatively activated macrophages to allergic lung inflammation: a tale of mice and men. J Innate Immun 4: 478–488. 22440980 10.1159/000336025 PMC6741623

[5] Sica A, Mantovani A (2012) Macrophage plasticity and polarization: in vivo veritas. J Clin Invest 122: 787–795. 22378047 10.1172/JCI59643 PMC3287223

[6] Yu H, Pardoll D, Jove R (2009) STATs in cancer inflammation and immunity: a leading role for STAT3. Nat Rev Cancer 9: 798–809. 19851315 10.1038/nrc2734 PMC4856025

[7] Mansell A, Smith R, Doyle SL, Gray P, Fenner JE, et al (2006) Suppressor of cytokine signaling 1 negatively regulates Toll-like receptor signaling by mediating Mal degradation. Nat Immunol 7: 148–155. 16415872 10.1038/ni1299

[8] Pelegrin P, Surprenant A (2009) Dynamics of macrophage polarization reveal new mechanism to inhibit IL-1beta release through pyrophosphates. EMBO J 28: 2114–2127. 19536133 10.1038/emboj.2009.163 PMC2699392

[9] Porta C, Rimoldi M, Raes G, Brys L, Ghezzi P, et al (2009) Tolerance and M2 (alternative) macrophage polarization are related processes orchestrated by p50 nuclear factor kappaB. Proc Natl Acad Sci U S A 106: 14978–14983. 19706447 10.1073/pnas.0809784106 PMC2736429

[10] Mantovani A, Sica A (2010) Macrophages, innate immunity and cancer: balance, tolerance, and diversity. Curr Opin Immunol 22: 231–237. 20144856 10.1016/j.coi.2010.01.009

[11] Pauleau AL, Rutschman R, Lang R, Pernis A, Watowich SS, et al (2004) Enhancer-mediated control of macrophage-specific arginase I expression. J Immunol 172: 7565–7573. 15187136 10.4049/jimmunol.172.12.7565

[12] Kortylewski M, Kujawski M, Wang T, Wei S, Zhang S, et al (2005) Inhibiting Stat3 signaling in the hematopoietic system elicits multicomponent antitumor immunity. Nat Med 11: 1314–1321. 16288283 10.1038/nm1325

[13] Ostrand-Rosenberg S, Grusby MJ, Clements VK (2000) Cutting edge: STAT6-deficient mice have enhanced tumor immunity to primary and metastatic mammary carcinoma. J Immunol 165: 6015–6019. 11086031 10.4049/jimmunol.165.11.6015

[14] Sinha P, Clements VK, Ostrand-Rosenberg S (2005) Reduction of myeloid-derived suppressor cells and induction of M1 macrophages facilitate the rejection of established metastatic disease. J Immunol 174: 636–645. 15634881 10.4049/jimmunol.174.2.636

[15] Odegaard JI, Ricardo-Gonzalez RR, Goforth MH, Morel CR, Subramanian V, et al (2007) Macrophage-specific PPARgamma controls alternative activation and improves insulin resistance. Nature 447: 1116–1120. 17515919 10.1038/nature05894 PMC2587297

[16] Liao X, Sharma N, Kapadia F, Zhou G, Lu Y, et al (2011) Kruppel-like factor 4 regulates macrophage polarization. J Clin Invest 121: 2736–2749. 21670502 10.1172/JCI45444 PMC3223832

[17] Sly LM, Rauh MJ, Kalesnikoff J, Song CH, Krystal G (2004) LPS-induced upregulation of SHIP is essential for endotoxin tolerance. Immunity 21: 227–239. 15308103 10.1016/j.immuni.2004.07.010

[18] An H, Xu H, Zhang M, Zhou J, Feng T, et al (2005) Src homology 2 domain-containing inositol-5-phosphatase 1 (SHIP1) negatively regulates TLR4-mediated LPS response primarily through a phosphatase activity- and PI-3K-independent mechanism. Blood 105: 4685–4692. 15701712 10.1182/blood-2005-01-0191

[19] Gabhann JN, Higgs R, Brennan K, Thomas W, Damen JE, et al (2010) Absence of SHIP-1 results in constitutive phosphorylation of tank-binding kinase 1 and enhanced TLR3-dependent IFN-beta production. J Immunol 184: 2314–2320. 20100929 10.4049/jimmunol.0902589

[20] O’Connell RM, Chaudhuri AA, Rao DS, Baltimore D (2009) Inositol phosphatase SHIP1 is a primary target of miR-155. Proceedings of the National Academy of Sciences 106: 7113–7118. 10.1073/pnas.0902636106 PMC2678424 19359473

[21] Spence S, Fitzsimons A, Boyd CR, Kessler J, Fitzgerald D, et al (2013) Suppressors of cytokine signaling 2 and 3 diametrically control macrophage polarization. Immunity 38: 66–78. 23177319 10.1016/j.immuni.2012.09.013

[22] Ni Gabhann J, Spence S, Wynne C, Smith S, Byrne JC, et al.. (2011) Defects in acute responses to TLR4 in Btk-deficient mice result in impaired dendritic cell-induced IFN-gamma production by natural killer cells. Clin Immunol. 10.1016/j.clim.2011.12.009 22281426

[23] Doyle SL, Jefferies CA, O’Neill LA (2005) Bruton’s tyrosine kinase is involved in p65-mediated transactivation and phosphorylation of p65 on serine 536 during NFkappaB activation by lipopolysaccharide. J Biol Chem 280: 23496–23501. 15849198 10.1074/jbc.C500053200

[24] Doyle SL, Jefferies CA, Feighery C, O’Neill LA (2007) Signaling by Toll-like receptors 8 and 9 requires Bruton’s tyrosine kinase. J Biol Chem 282: 36953–36960. 17932028 10.1074/jbc.M707682200

[25] Horwood NJ, Mahon T, McDaid JP, Campbell J, Mano H, et al (2003) Bruton’s tyrosine kinase is required for lipopolysaccharide-induced tumor necrosis factor alpha production. J Exp Med 197: 1603–1611. 12810683 10.1084/jem.20021845 PMC2193950

[26] Horwood NJ, Page TH, McDaid JP, Palmer CD, Campbell J, et al (2006) Bruton’s tyrosine kinase is required for TLR2 and TLR4-induced TNF, but not IL-6, production. J Immunol 176: 3635–3641. 16517732 10.4049/jimmunol.176.6.3635

[27] Lee KG, Xu S, Kang ZH, Huo J, Huang M, et al (2012) Bruton’s tyrosine kinase phosphorylates Toll-like receptor 3 to initiate antiviral response. Proc Natl Acad Sci U S A 109: 5791–5796. 22454496 10.1073/pnas.1119238109 PMC3326448

[28] Krausgruber T, Blazek K, Smallie T, Alzabin S, Lockstone H, et al (2011) IRF5 promotes inflammatory macrophage polarization and TH1-TH17 responses. Nat Immunol 12: 231–238. 21240265 10.1038/ni.1990

[29] Fleetwood AJ, Dinh H, Cook AD, Hertzog PJ, Hamilton JA (2009) GM-CSF- and M-CSF-dependent macrophage phenotypes display differential dependence on Type I interferon signaling. Journal of Leukocyte Biology 86: 411–421. 19406830 10.1189/jlb.1108702

[30] Hendriks RW, de Bruijn MF, Maas A, Dingjan GM, Karis A, et al (1996) Inactivation of Btk by insertion of lacZ reveals defects in B cell development only past the pre-B cell stage. EMBO J 15: 4862–4872. 8890160 PMC452224

[31] Livak KJ, Schmittgen TD (2001) Analysis of relative gene expression data using real-time quantitative PCR and the 2(-Delta Delta C(T)) Method. Methods 25: 402–408. 11846609 10.1006/meth.2001.1262

[32] Smith P, Mangan NE, Fallon PG (2009) Generation of parasite antigens for use in Toll-like receptor research. Methods Mol Biol 517: 401–413. 19378020 10.1007/978-1-59745-541-1_24

[33] Mangan NE, Dasvarma A, McKenzie AN, Fallon PG (2007) T1/ST2 expression on Th2 cells negatively regulates allergic pulmonary inflammation. Eur J Immunol 37: 1302–1312. 17407196 10.1002/eji.200636520

[34] Hams E, Saunders SP, Cummins EP, O’Connor A, Tambuwala MT, et al (2011) The hydroxylase inhibitor dimethyloxallyl glycine attenuates endotoxic shock via alternative activation of macrophages and IL-10 production by B1 cells. Shock 36: 295–302. 21844787 10.1097/SHK.0b013e318225ad7e PMC3157050

[35] Ghosn EE, Cassado AA, Govoni GR, Fukuhara T, Yang Y, et al (2010) Two physically, functionally, and developmentally distinct peritoneal macrophage subsets. Proc Natl Acad Sci U S A 107: 2568–2573. 20133793 10.1073/pnas.0915000107 PMC2823920

[36] Taneichi H, Kanegane H, Sira MM, Futatani T, Agematsu K, et al (2008) Toll-like receptor signaling is impaired in dendritic cells from patients with X-linked agammaglobulinemia. Clin Immunol 126: 148–154. 18271077 10.1016/j.clim.2007.10.005

[37] Gordon S (2003) Alternative activation of macrophages. Nat Rev Immunol 3: 23–35. 12511873 10.1038/nri978

[38] Lin SL, Castaño AP, Nowlin BT, Lupher ML, Duffield JS (2009) Bone Marrow Ly6Chigh Monocytes Are Selectively Recruited to Injured Kidney and Differentiate into Functionally Distinct Populations. The Journal of Immunology 183: 6733–6743. 19864592 10.4049/jimmunol.0901473

[39] Mantovani A, Sica A, Sozzani S, Allavena P, Vecchi A, et al (2004) The chemokine system in diverse forms of macrophage activation and polarization. Trends Immunol 25: 677–686. 15530839 10.1016/j.it.2004.09.015

[40] Martinez FO, Gordon S, Locati M, Mantovani A (2006) Transcriptional profiling of the human monocyte-to-macrophage differentiation and polarization: new molecules and patterns of gene expression. J Immunol 177: 7303–7311. 17082649 10.4049/jimmunol.177.10.7303

[41] Porcheray F, Viaud S, Rimaniol AC, Leone C, Samah B, et al (2005) Macrophage activation switching: an asset for the resolution of inflammation. Clin Exp Immunol 142: 481–489. 16297160 10.1111/j.1365-2249.2005.02934.x PMC1809537

[42] Akira S, Takeda K (2004) Toll-like receptor signalling. Nat Rev Immunol 4: 499–511. 15229469 10.1038/nri1391

[43] Ramalingam TR, Reiman RM, Wynn TA (2005) Exploiting worm and allergy models to understand Th2 cytokine biology. Curr Opin Allergy Clin Immunol 5: 392–398. 16131912 10.1097/01.all.0000182542.30100.6f

[44] Reese TA, Liang HE, Tager AM, Luster AD, Van Rooijen N, et al (2007) Chitin induces accumulation in tissue of innate immune cells associated with allergy. Nature 447: 92–96. 17450126 10.1038/nature05746 PMC2527589

[45] Perez de Diego R, Lopez-Granados E, Pozo M, Rodriguez C, Sabina P, et al (2006) Bruton’s tyrosine kinase is not essential for LPS-induced activation of human monocytes. J Allergy Clin Immunol 117: 1462–1469. 16751014 10.1016/j.jaci.2006.01.037

[46] Gagliardi MC, Finocchi A, Orlandi P, Cursi L, Cancrini C, et al (2003) Bruton’s tyrosine kinase defect in dendritic cells from X-linked agammaglobulinaemia patients does not influence their differentiation, maturation and antigen-presenting cell function. Clin Exp Immunol 133: 115–122. 12823285 10.1046/j.1365-2249.2003.t01-1-02178.x PMC1808743

[47] Jyonouchi H, Geng L, Toruner GA, Vinekar K, Feng D, et al (2008) Monozygous twins with a microdeletion syndrome involving BTK, DDP1, and two other genes; evidence of intact dendritic cell development and TLR responses. Eur J Pediatr 167: 317–321. 17520285 10.1007/s00431-007-0493-0

[48] Amoras AL, da Silva MT, Zollner RL, Kanegane H, Miyawaki T, et al (2007) Expression of Fc gamma and complement receptors in monocytes of X-linked agammaglobulinaemia and common variable immunodeficiency patients. Clin Exp Immunol 150: 422–428. 17900300 10.1111/j.1365-2249.2007.03512.x PMC2219363

[49] Liu X, Zhan Z, Li D, Xu L, Ma F, et al (2011) Intracellular MHC class II molecules promote TLR-triggered innate immune responses by maintaining activation of the kinase Btk. Nat Immunol 12: 416–424. 21441935 10.1038/ni.2015

[50] Li Q, Verma IM (2002) NF-kappaB regulation in the immune system. Nat Rev Immunol 2: 725–734. 12360211 10.1038/nri910

[51] Hu X, Li WP, Meng C, Ivashkiv LB (2003) Inhibition of IFN-gamma signaling by glucocorticoids. J Immunol 170: 4833–4839. 12707366 10.4049/jimmunol.170.9.4833

[52] Li X, Tupper JC, Bannerman DD, Winn RK, Rhodes CJ, et al (2003) Phosphoinositide 3 kinase mediates Toll-like receptor 4-induced activation of NF-kappa B in endothelial cells. Infect Immun 71: 4414–4420. 12874320 10.1128/IAI.71.8.4414-4420.2003 PMC166052

[53] Kitaura J, Asai K, Maeda-Yamamoto M, Kawakami Y, Kikkawa U, et al (2000) Akt-dependent cytokine production in mast cells. J Exp Med 192: 729–740. 10974038 10.1084/jem.192.5.729 PMC2193272

[54] Mohammad DK, Nore BF, Hussain A, Gustafsson MO, Mohamed AJ, et al (2013) Dual Phosphorylation of Btk by Akt/Protein Kinase B Provides Docking for 14-3-3zeta, Regulates Shuttling, and Attenuates both Tonic and Induced Signaling in B Cells. Mol Cell Biol 33: 3214–3226. 23754751 10.1128/MCB.00247-13 PMC3753922

[55] Takahashi-Tezuka M, Hibi M, Fujitani Y, Fukada T, Yamaguchi T, et al (1997) Tec tyrosine kinase links the cytokine receptors to PI-3 kinase probably through JAK. Oncogene 14: 2273–2282. 9178903 10.1038/sj.onc.1201071

[56] Uckun F, Ozer Z, Vassilev A (2007) Bruton’s tyrosine kinase prevents activation of the anti-apoptotic transcription factor STAT3 and promotes apoptosis in neoplastic B-cells and B-cell precursors exposed to oxidative stress. Br J Haematol 136: 574–589. 17367410 10.1111/j.1365-2141.2006.06468.x

[57] Ohmori Y, Hamilton TA (2001) Requirement for STAT1 in LPS-induced gene expression in macrophages. J Leukoc Biol 69: 598–604. 11310846

[58] Rauh MJ, Ho V, Pereira C, Sham A, Sly LM, et al (2005) SHIP represses the generation of alternatively activated macrophages. Immunity 23: 361–374. 16226502 10.1016/j.immuni.2005.09.003

[59] Antignano F, Ibaraki M, Kim C, Ruschmann J, Zhang A, et al (2010) SHIP is required for dendritic cell maturation. J Immunol 184: 2805–2813. 20154203 10.4049/jimmunol.0903170

[60] Weisser SB, McLarren KW, Voglmaier N, van Netten-Thomas CJ, Antov A, et al (2011) Alternative activation of macrophages by IL-4 requires SHIP degradation. Eur J Immunol 41: 1742–1753. 21469115 10.1002/eji.201041105 PMC6902421

[61] Arranz A, Doxaki C, Vergadi E, Martinez de la Torre Y, Vaporidi K, et al (2012) Akt1 and Akt2 protein kinases differentially contribute to macrophage polarization. Proc Natl Acad Sci U S A 109: 9517–9522. 22647600 10.1073/pnas.1119038109 PMC3386059

[62] Xu F, Kang Y, Zhang H, Piao Z, Yin H, et al (2013) Akt1-Mediated Regulation of Macrophage Polarization in a Murine Model of Staphylococcus aureus Pulmonary Infection. J Infect Dis 208: 528–538. 23613163 10.1093/infdis/jit177

[63] Taha RA, Minshall EM, Miotto D, Shimbara A, Luster A, et al (1999) Eotaxin and monocyte chemotactic protein-4 mRNA expression in small airways of asthmatic and nonasthmatic individuals. J Allergy Clin Immunol 103: 476–483. 10069883 10.1016/s0091-6749(99)70474-4

[64] Kawakami Y, Inagaki N, Salek-Ardakani S, Kitaura J, Tanaka H, et al (2006) Regulation of dendritic cell maturation and function by Bruton’s tyrosine kinase via IL-10 and Stat3. Proc Natl Acad Sci U S A 103: 153–158. 16371463 10.1073/pnas.0509784103 PMC1325006

[65] Hata D, Kawakami Y, Inagaki N, Lantz CS, Kitamura T, et al (1998) Involvement of Bruton’s tyrosine kinase in FcepsilonRI-dependent mast cell degranulation and cytokine production. J Exp Med 187: 1235–1247. 9547335 10.1084/jem.187.8.1235 PMC2212237

[66] Shabestari MS, Rezaei N (2008) Asthma and allergic rhinitis in a patient with BTK deficiency. J Investig Allergol Clin Immunol 18: 300–304. 18714539

[67] Costa-Carvalho BT, Wandalsen GF, Pulici G, Aranda CS, Sole D (2011) Pulmonary complications in patients with antibody deficiency. Allergol Immunopathol (Madr) 39: 128–132. 21339034 10.1016/j.aller.2010.12.003

[68] D’Cruz OJ, Uckun FM (2013) Novel Bruton’s tyrosine kinase inhibitors currently in development. Onco Targets Ther 6: 161–176. 23493945 10.2147/OTT.S33732 PMC3594038

[69] Honigberg LA, Smith AM, Sirisawad M, Verner E, Loury D, et al (2010) The Bruton tyrosine kinase inhibitor PCI-32765 blocks B-cell activation and is efficacious in models of autoimmune disease and B-cell malignancy. Proc Natl Acad Sci U S A 107: 13075–13080. 20615965 10.1073/pnas.1004594107 PMC2919935

[70] Byrne JC, Ní Gabhann J, Stacey K, Coffey BM, McCarthy E, et al.. (2013) Bruton’s Tyrosine Kinase Is Required for Apoptotic Cell Uptake via Regulating the Phosphorylation and Localization of Calreticulin. J Immunol In press. 10.4049/jimmunol.1300057 23596312

